# Hydroxycobalamin catalyzes the oxidation of diethyldithiocarbamate and increases its cytotoxicity independently of copper ions

**DOI:** 10.1016/j.redox.2018.09.016

**Published:** 2018-09-25

**Authors:** M.E. Solovieva, Yu.V. Shatalin, V.V. Solovyev, A.V. Sazonov, V.P. Kutyshenko, V.S. Akatov

**Affiliations:** a Institute of Theoretical and Experimental Biophysics, Russian Academy of Sciences, Pushchino, Moscow Region 142290 Russia; b JSC BIOCAD, Saint-Petersburg 198515, Russia

**Keywords:** HOCbl, hydroxycobalamin (a form of vitamin В_12_), Cbl, cobalamin, DDC, N,N- diethyldithiocarbamate, DTC, dithiocarbamates, DSF, disulfiram, SOD, superoxide dismutase, ROS, reactive oxygen species, BCS, bathocuproine disulfonate, NAC, N-acetylcysteine, GSH, glutathione, HBSS, Hank's solution, LC/MS, mass spectrometry, RT, retention time, Diethyldithiocarbamate, Disulfiram, Hydroxycobalamin, Vitamin B_12_, Oxidative stress, Cytotoxicity

## Abstract

It is known that some metals (Cu, Zn, Cd, Au) markedly increase the toxic effect of thiocarbamates. It was shown in the present study that hydroxycobalamin (a form of vitamin B_12_, HOCbl), which incorporates cobalt, significantly enhances the cytotoxicity of diethyldithiocarbamate (DDC), decreasing its IC_50_ value in tumor cells three to five times. The addition of HOCbl to aqueous DDC solutions accelerated the reduction of oxygen. No hydrogen peroxide accumulation was observed in DDC + HOCbl solutions; however, catalase slowed down the oxygen reduction rate. Catalase as well as the antioxidants N-acetylcysteine (NAC) and glutathione (GSH) partially inhibited the cytotoxic effect of DDC + HOCbl, whereas ascorbate, pyruvate, and tiron, a scavenger of superoxide anion, had no cytoprotective effect. The administration of HOCbl into DDC solutions (> 1 mM) resulted in the formation of a crystalline precipitate, which was inhibited in the presence of GSH. The data of UV and NMR spectroscopy and HPLC and Mass Spectrometry (LC/MS) indicated that the main products of the reaction of DDC with HOCbl are disulfiram (DSF) and its oxidized forms, sulfones and sulfoxides. The increase in the cytotoxicity of DDC combined with HOCbl occurred both in the presence of Cu^2+^ in culture medium and in nominally Cu-free solutions, as well as in growth medium containing the copper chelator bathocuproine disulfonate (BCS). The results indicate that HOCbl accelerates the oxidation of DDC with the formation of DSF and its oxidized forms. Presumably, the main cause of the synergistic increase in the toxic effect of DDC + HOCbl is the formation of sulfones and sulfoxides of DSF.

## Highlights

• HOCbl synergistically enhances the cytotoxicity of DDC against tumor cells.
• HOCbl catalyzes the oxidation of DDC and the formation of DSF and its oxidized forms.
• HOCbl enhances DDC cytotoxicity by catalyzing extracellular DSF oxidation.
• HOCbl catalyzes DSF oxidation in DDC solutions in a radical-mediated manner.
• HOCbl enhances DDC cytotoxicity independently of extracellular Cu ions.

## Graphical abstract

**Figure:**
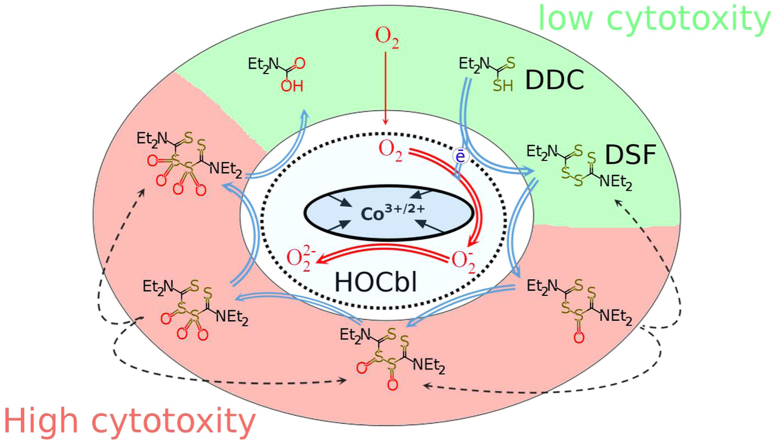

## 1 Introduction

SH-containing compounds are involved in the scavenging of reactive oxygen species (ROS) and are widely used as antioxidants. In reactions with ions of transition metals, thiols are capable of generating ROS and producing a damaging action on cells and tissues [1], [2], [3], [4]. In addition, in reactions with free radicals, they can transform into thiyl radicals, which can lead to the damage to DNA and other biomolecules [5]. Jacobsen et al. (1984) showed that cobalamin (Cbl) derivatives catalyze the aerobic oxidation of the thiols 2-mercaptoethanol and dithioerythritol, which results in the formation of disulfides and hydrogen peroxide [6]. We have previously shown that hydroxycobalamin (a form of vitamin B_12_, HOCbl), in combination with the antioxidant thiols GSH, NAC, and DTT is capable of catalyzing the formation of hydrogen peroxide at concentrations up to 30–250 μM. This catalysis considerably enhances the cytotoxic action of these classical thiol compounds and leads to the manifestation of the prooxidant action of DTT, GSH, and NAC [7], [8].

Another interesting and widely used group of SH-containing compounds is dithiocarbamates (DTC). Compounds based on DTC are widely used in industry, veterinary science, agronomy, and medicine [9], [10]. One of the widely known carbamates is diethyldithiocarbamate (DDC), a dithio derivative of diethylcarbamic acid. DDC at millimolar concentrations (0,5-1 мМ) is used as a Cu,Zn-SOD inhibitor, which produces a prooxidant effect; however, under some conditions, it exhibits antioxidant and antiapoptotic properties [11], [12]. Under in vivo conditions, DDC appears in the bloodstream as a result of the metabolism of the anti-alcohol drug disulfiram (DSF) and undergoes further transformations in the liver and kidneys [13], [14], [15]. There is substantial evidence in the literature indicating that, due to the binding of copper ions, DTC produce a pronounced cytotoxic effect [13], [14], [16], [17], [18]. DTC are also capable of forming complexes with zinc, cadmium, and gold; at present, their antineoplastic activity is extensively studied [19], [20], [21], [22], [23]. It was shown that the chelation of copper by DDC/DSF and its transport to the cell leads to the generation of intracellular ROS [24], [25], [26], [27], [28]. To date, attempts have been made to introduce DSF and its derivatives into antitumor therapy since it was found that they synergistically enhance the cytotoxic effect of antineoplastic drugs, such as cisplatin, gemcitabine, and paclitaxel and increase the efficacy of some therapeutic methods, e.g., radiotherapy [20], [26], [28], [29], [30], [31], [32], [33]. It was found that the active metabolite of DSF when it is used in combination with copper is a dithiocarb-copper complex [17], [34]. Because this complex can damage normal tissues, different ways to increase the DSF toxicity without the use of copper ions and to protect the adjacent tissues by encapsulating the components into micro- and nanoparticles are developed to date [15], [35], [36].

We have found earlier that HOCbl is capable of enhancing the cytotoxic effect of DDC. The mechanism of this effect is not yet clearly established; however it is known that cobalt ions do not significantly affect the toxicity of DDC and DSF [17], [22]. Cobalamins form complexes with a variety of biologically active substances, participate as cofactors in many biological processes in the organism, and affect gene expression [37], [38]. The enhancement of the cytotoxic effect of DDC by Cbl detected in the study may be taken as the basis in the design of novel antitumor drugs. The use of modern ways of isolating active components by incapsulation (incorporation into liposomes as well as micro- and nanoparticles) [15], [36], [39], [40], [41] will enable one to avoid a possible unfavorable effect of the combination of DDC with Cbl on normal tissues. The goal of this work was to establish the mechanism by which HOCbl enhances the cytotoxic effect of DDC. We examined the products obtained during the reaction in solutions (DMEM, HBSS) and the cytotoxic effect of DDC + HOCbl in a culture of human tumor cells. We found that HOCbl catalyzes the aerobic oxidation of SH groups followed by DSF precipitation. In the aqueous phase, the accumulation of oxidized DSF derivatives occurs, which just causes the cytotoxic effect. Thus, under in vitro conditions, DDC+HOCbl is a binary catalytic system with a prolonged toxic effect.

## 2 Materials and methods

### 2.1 Chemicals

DDC, GSH and NAC were purchased from MPbiomedicals (USA); fetal bovine serum was from Gibco (USA). Acetone d6 (99.96%) was from CIL (UK). Other chemicals were from Sigma (USA).

### 2.2 Cell culture

Human lung carcinoma A549, human epidermoid larynx carcinoma HEp-2, and human squamous carcinoma A431 cell lines were obtained from the Russian Cell Culture Collection (Institute of Cytology, Russian Academy of Sciences, St. Petersburg). Cells were grown in DMEM supplemented with 10% FBS, 80 mg/l of gentamycin, and 20 mM sodium bicarbonate at 37 °C in an atmosphere of 5% CO_2_.

### 2.3 Cytotoxicity assay and treatment of cells

Сells were seeded in 96-well microplates or culture dishes (Corning, USA) at a concentration of 2 × 10^5^ cells/ml (2 × 10^4^ cells in 100 μl/well). DDC was added from freshly prepared stock solutions (10–100 mM) in deionized H_2_O, PBS, and HBSS, and HOCbl was added from a 2.5 mM stock solution in deionized H_2_O. All treatments were made 24 h after cell seeding. DSF was added from the stock solution (200 mM) in DMSO under continuous stirring to a concentration of 200 μM and below. Cytotoxicity was determined using the crystal violet assay as described earlier [8]. Cell viability was also estimated by the trypan blue exclusion assay.

### 2.4 Detection of hydrogen peroxide in culture medium

Oxygen reduction by DDC and HOCbl in PBS was estimated in a 1-ml chamber with an O_2_-electrode at 25 °C by polarography [8].

### 2.5 Estimation of DSF solubility

From a solution of DSF in DMSO, calibrating solutions in PBS, DMEM, and DMEM + serum were prepared through a series of intermediate dilutions at a DMSO concentration of 1%. The solutions were allowed to stand for 24 h at 37 ^о^С in an atmosphere of 5% СО_2_. Then, the samples were centrifuged (5 min, 14,500 rpm), and UV spectra of supernatants were recorded on a Cary 100 Scan spectrophotometer (Varian, Australia). The limit of DSF solubility was determined by extrapolating the linear segment of the dependence D = f(C) onto the region of optical densities of saturated DSF solutions.

### 2.6 UV spectrophotometry

Absorption spectra were measured in PBS on a Cary 100 Scan spectrophotometer. If necessary, the reaction mixture was diluted with PBS (1:10).

### 2.7 NMR spectroscopy

The main product of the reaction of HOCbl with DDC was identified on an AVANCE-III 600 spectrophotometer (Bruker, Germany) with a working frequency of 600 MHz. The temperature of a sample was 298 К. The spectrum width was 23.4 ppm. The duration of an impulse was 10 μs. For a good signal/noise ratio, no more than 32 scans were required. The repetition time was 1.14 s. The relaxation delay between scans was 10 s in the mode of simple scanning, without the suppression of signals of the solvent. All samples were dissolved in acetone.

### 2.8 HPLC and mass spectrometry

LC/MS was performed using a Waters Aquity UPLC system connected in photodiode array detector followed by a LCQ Deca XP (Thermo Finnigan, USA) mass spectrometer operating in the electrospray ionization mode.

The substances were separated using the following conditions and parameters of mass spectrometry. For HOCbl complexes, gradient chromatography was performed at 25 °С using a Synergi Hydro-RP 80 A column (4 µm, 4.6 × 50 mm) at a flow rate of 1.0 ml/min using water (solvent A) and CH3CN (solvent B). Positive electrospray ionization was used. Other LC/MS parameters were as follows: capillary voltage 4.50 kV and cone voltage 80 V. The source temperature was 250 °С. For the separation and detection of low-molecular-weight compounds, chromatography was performed at 25 °С using a Symmetry C18 column (5 µm, 4.6 × 150 mm) at a flow rate of 1.0 ml/min using the isocratic elution mode water/methanol (20/80 by volume). Positive electrospray ionization was used. Other LC/MS parameters were as follows: capillary voltage 4.00 kV and cone voltage 80 V. The source temperature was 165 °С.

All data were collected and processed using the Xcalibur® software. Samples were prepared for analysis by adding HOCbl to a PBS containing DDC with gentle stirring followed by filtration of the sample and dilution with water (1: 20).

### 2.9 Statistical analysis

Each experiment was performed at least three times. All the values represent the means ± s.e.m. The statistical significance of the results was analyzed using the Student's *t*-test for paired experiments. The values of *P* < 0.05 were considered as statistically significant.

## 3 Results

### 3.1 HOCbl enhances the cytotoxic effect of DDC

**Fig. 1:**
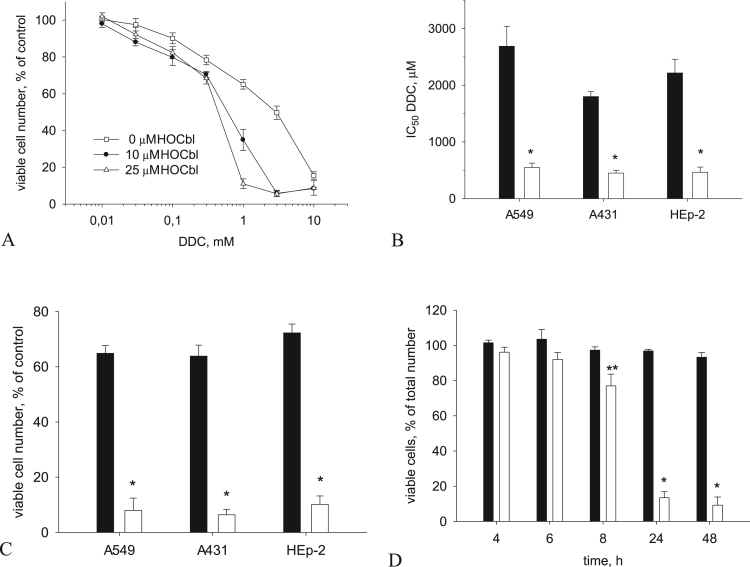
Enhancement of the cytotoxic effect of DDC by HOCbl. (А). Effect of the concentration of HOCbl and DDС on the viability of А549 cells. (B). A decrease in the IC_50_ value for DDC in combination with 25 μM HOCbl in НЕр–2, А431 and А549 cells. (С). Cytotoxic effect of 48-h incubation of НЕр–2, А431, and А 549 cells with DDC + HOCbl. (D). The time course of the death of А549 cells in the presence of DDC + HOCbl. The components (25 μM HOCbl and 1 mM DDC) were added simultaneously 24 h after the seeding of cells. The action of DDC + HOCbl was interrupted by replacing the culture medium with a fresh growth medium. The cytotoxicity was estimated at 48 h after the addition of the components (see Materials and methods). The data are the means ± SEM of five separate experiments. Dark columns denote 1 mM DDC, and light columns denote DDC + HOCbl. *P < 0.01, **P < 0.05 compared to cells treated with 1 mM DDC.

DDC in the range of concentrations less than 1 mM did not affect the viability of А549, НЕр–2, and А431 cells over a period of 48 h, which agrees well with the results reported by other authors for pyrrolidine dithiocarbamate, an analog of DDC [19], [42]. During the incubation of cells with 1 mM DDC, a minor cytostatic effect was observed: the total number of cells in a population decreased by 48 h to 65–75% compared to the untreated control, but the number of dead cells was the same as in the control, no higher than 5% (Fig. 1). At DDC concentrations above 1 mM, the amount of viable cells decreased (Fig. 1А). The IC_50_ value for DDC in the cells used was 1.6–2.6 mM (Fig. 1В). HOCbl at a concentration of 10 μM and above enhanced the cytotoxic effect of DDC, which was maximum at 25 μM of HOCbl (Fig. 1А). The IC_50_ values for DDC in combination with 25 μM HOCbl were 0.45–0.6 mM (Fig. 1В). We found that, after 6 h of incubation with 1 mM DDC and 25 μM HOCbl (hereafter referred to as DDC + HOCbl), the number of dead cells significantly increased (Fig. 1D), and 48- h incubation led to the death of 90–95% of cells (Figs. 1С, 1D). HOCbl administered alone at concentrations up to 3 mM was not toxic.

### 3.2 Role of exogenous oxidative stress in the enhancement of the cytotoxicity of DDC by HOCbl

**Fig. 2:**
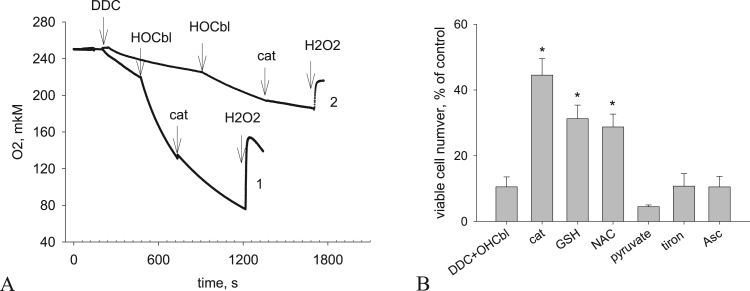
Oxidative stress induced by the combined action of DDC and HOCbl. (A). HOCbl (25 μM) catalyzes the reduction of oxygen by DDC. Oxygen concentration was measured after the addition of DDC and HOCbl to PBS in a chamber at 20 °C using an O_2_ electrode. Curve *1*: 10 mM DDC, 250 μM HOCbl, 500 U catalase, 150 μM H_2_O_2_. Сurve *2*: 1 mM DDC, 25 μM HOCbl, 100 U catalase, 50 μM H_2_O_2_. (В). Antioxidants partially inhibit the cytotoxic effect induced by DDC + HOCbl in А549 cells. Antioxidants were added to the cells 24 h after the seeding in 96-well plates, 1 h prior to, or simultaneously with the addition of 1 mM DDC + 25 μM HOCbl. The action of DDC + HOCbl was interrupted by replacing the culture medium with a fresh growth medium. The cytotoxicity was estimated at 48 h after the addition of the components (see Materials and methods). The data are the means ± SEM of five separate experiments. * *P* < 0.01, compared to cells treated with DDC + HOCbl.

We found that DDC reduces dissolved oxygen in a dose-dependent manner (Fig. 2A): 1 mM DDC induced oxygen uptake with an average rate of 1.66 ± 0.25 μM/min, whereas the oxygen uptake in the presence of 10 mM DDC increased three- to fourfold, to 5.64 ± 0.60 μM/min (n = 6). The addition of 250 μM HOCbl accelerated oxygen reduction on the average to 13.94 ± 2.04 μM/min (n = 6). Catalase (500–1000 U) did not increase the concentration of oxygen in the solution, indicating that there was no accumulation of hydrogen peroxide. However, catalase significantly slowed down the oxygen reduction, to 7.88 ± 0.77 μM/min. This indicates that the reaction of DDC with HOCbl results in the generation of hydrogen peroxide, which is quickly reduced in the further reaction with the formation of an oxidized product. Note that the addition of SOD did not affect the rate of oxygen reduction in DDC solutions (not shown).

We estimated the effect of catalase and other antioxidants on the cytotoxic action of the combination. Catalase (200 U/ml) partially prevented the cell death (Fig. 2B); the amount of survived cells increased to 30–40%. Ascorbic acid (Asc, 500 μM), pyruvate (4–10 mM), and the cell-permeable superoxide scavenger tiron (0.1–1 mM) did not inhibit the cytotoxic effect of the DDC + HOCbl. After the addition of NAC and GSH (10 mM each), the death induced by the DDC + HOCbl was partially inhibited in a dose-dependent manner (Fig. 2B).

### 3.3 Study of the products of reaction between DDC and HOCbl

#### 3.3.1 Study of the major poorly soluble reaction product

We found that, at DDC concentrations more than 1 mM, the reaction of DDC with HOCbl was accompanied by the dose-dependent precipitation of crystals which was observed even 20–30 min after the addition of HOCbl to DDC and continued for several hours. The precipitation of crystals occurred in deionized water, PBS, HBSS, and in culture medium with and without serum. The form of crystals was affected by the composition of medium, as well as the presence and concentration of catalase. In the presence of GSH and NAC (10 mM and higher), the formation of crystals was inhibited. These observations suggest that crystal formation in the reaction of DDC with HOCbl is a redox-dependent process.

**Fig. 3:**
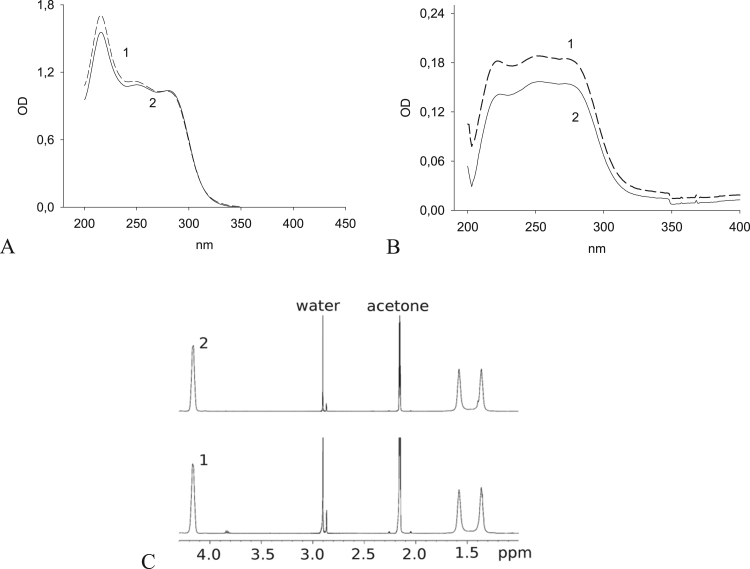
UV and NMR spectra of a sediment (1) and commercial DSF (2) dissolved in ethanol (А), PBS (pH 7.2) (В), and acetone (С). The sediment was obtained in a solution of 10 mM DDC and 250 μM HOCbl in deionized water. The concentration of the crystalline sediment is 30 mg/l in alcohol and 6 mg/l in PBS, and the concentration of commercial DSF is 30 mg/l and 4.25 mg/l, respectively.

We assumed that the resulting crystalline substance is DSF. The limit of its solubility, determined by photometry of a saturated solution of the substance at 37 °С 24 h after the preparation of the solution, is 68.87 ± 4.75 μM (n = 4) and coincides with the solubility of commercial DSF. Fig. 3 shows a UV spectrum of the precipitate, which is identical to the spectrum of commercial DSF and has characteristic absorption maxima in PBS at 226, 256, and 276 nm (Figs. 3A and 3B, respectively, in alcohol and PBS).

Fig. 3С shows ^1^H NMR spectra of commercial DSF and of the acetone-soluble crystalline precipitate obtained in the reaction of 10 mM DDC with 250 μM HOCbl. The examination of the precipitate by COSY and NOE spectroscopy led us to conclude that it is a dimer of DDC. As can be seen in Fig. 3С, its spectrum is identical to the spectrum of commercial DSF (Sigma). The signals of the methylene groups of the DDC dimer at ~1.6 and ~1.35 ppm indicate that they can exist in two slightly different conformations. The signal at ~4.17 ppm belongs to the DDC methylene group, which exists in one conformation. Thus, the results of UV and NMR spectroscopy indicate that precipitated crystals are DSF.

#### 3.3.2 Spectrophotometric examination of the kinetics of the reaction of DDC with HOCbl

**Fig. 4:**
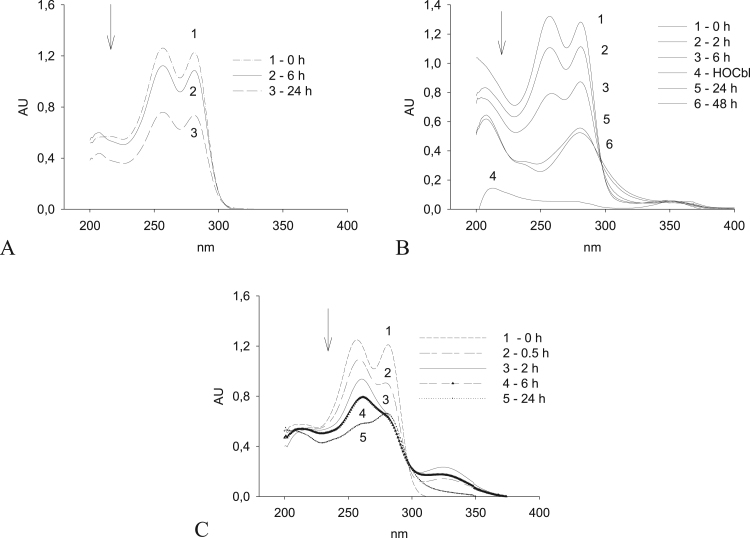
Changes in UV spectra of aqueous solutions of 1 mM DDC (А), 1 mM DDC combined with 25 μM HOCbl (В), 0.1 mM DDC + 0.1 mM Н_2_О_2_ (С). (А, В). Before measurements, DDC and DDC + HOCbl solutions were diluted tenfold; only the spectra of unilluminated solutions were recorded.

The absorption spectra of DDC solutions in PBS (pH 7.4, 37 °C) show maxima at 256 and 280 nm. As seen from Fig. 4А, the amplitude of the peaks decreases by an equivalent value, indicating a decrease in DDC concentration.

Within the first minutes of the oxidation of DDC in the presence of HOCbl, a clearly pronounced absorption occurs in the region of 200–210 nm, which may indicate the formation of a DDC–Cbl complex. According to the literature data, absorption recorded at 210 nm is characteristic, in particular, also of sulfoxides. Over a period of 48 h, the absorbance at 210 nm decreases compared with the initial value; however, the intensity of the peak at 210 nm increases compared to the intensity of peaks at 280 and, especially, at 256 nm. The intensity of the peaks at 256 and 280 nm changes asynchronously throughout the observation period (24 h), with the strongest decrease being observed at 256 nm (Fig. 4B).

It has been found earlier that the oxidation of thiols in the presence of HOCbl is accompanied by the generation of hydrogen peroxide [6], [7], [8]. In addition, there is evidence that DDC can be oxidized by H_2_O_2_ [43]. However, the changes in the spectra within the first 6 h of the reaction between DDC and HOCbl (Fig. 4B) differed from those in the reaction of DDC with H_2_O_2_ (Fig. 4C) and were rather similar to spectral changes in DDC solutions irradiated by gamma rays and oxidized by free-radical mechanisms [43].

#### 3.3.3 LC/MS аnalysis of the reaction of DDC with HOCbl

**Fig. 5:**
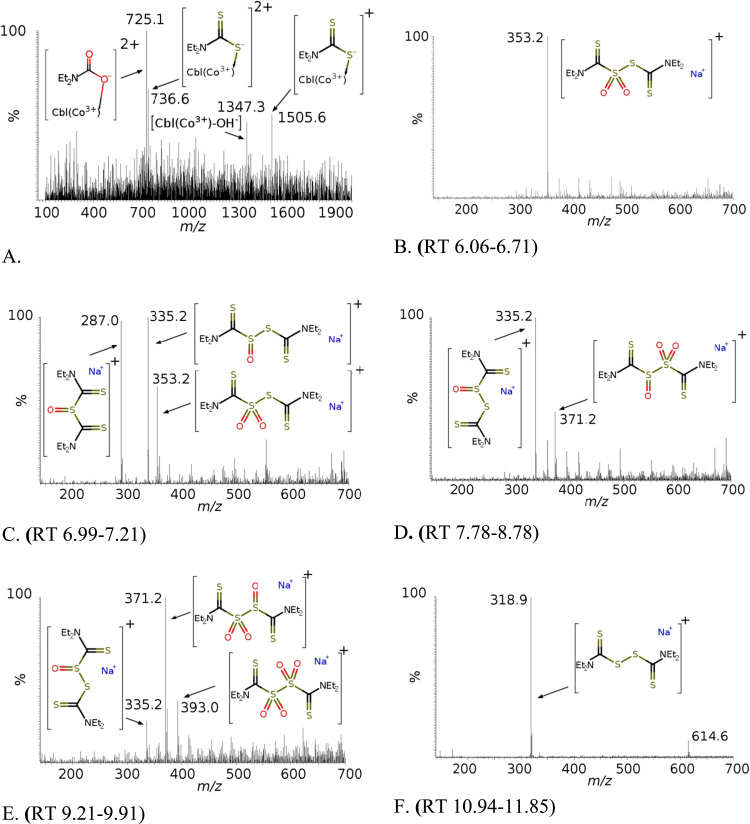
A mass spectrum of DDC–Cbl (A) and low-molecular-weight products of DDC oxidation recorded after 4 h of the reaction (B–F).

An analysis of the products of the reaction between DDC and HOCbl in PBS by LC/MS revealed some products that had different RT and different *m/z* values. After 30 min of incubation of DDC + HOCbl, along with signals from [Cbl+Na^+^]^+^ (*m/z* 1347.3), the spectrum showed peaks of the complexes of HOCbl with DDC [Cbl–DDC+Na^+^]^+^ (*m/z* 1505.6; 736.6) and with N,N-diethylcarbamic acid (*m/z* 725.1), which resulted from the complete oxidation of DDC (Fig. 5A). A signal with the *m/z* value corresponding to the Cbl–DSF complex was not detected, which indicates either a rapid dissociation of this intermediate or the absence of marked binding of DSF to Cbl.

Based on the fact that sulfur can oxidize to sulfoxide and sulfonic derivatives as a result of oxidation of disulfide compounds [44], [45], we assumed that the oxidized compounds are formed in the medium simultaneously with the main product (DSF). Four hours after the onset of the reaction of DDC with HOCbl, four main low-molecular products were separated by chromatography. The main product, a product with the maximum concentration, was DSF ([DSF+Na^+^]^+^, *m/z* 318.9; [2DSF+Na^+^, *m/z* 615.2]^+^, RT 10.94–11.85) (Fig. 5F). Two reaction products, which showed peaks of close intensities upon chromatographic separation, correspond probably to a thiosulfonate derivative of DSF (Et2NC(=S)S(=O)_2_SC(= S)NEt2) (RT = 5.4–6.7, *m/z* 353.2) (Fig. 5B) and a thiosulfinate derivative of DSF (Et2NC(= S)SS(= O)C(= S)NEt2) (RT = 7.0–7.2, *m/z* 335.2) (Fig. 5C). The thiosulfinate derivative of DSF is rather unstable and is transformed as a result of the exchange reaction into a thiosulfonate derivative and monosulfiram, which are identified together with the main product.

The minor product resulting from the chromatographic separation of the mixture corresponds probably to sulfinyl sulfone (*m/z* 371.2) recorded at RT = 9.21–9.91 (Fig. 5D). This product is unstable and disproportionates already upon separation into a disulfoxide (*m/z* 335.2) (Fig. 5D,E) and a disulfonic derivative of DSF (*m/z* 393.0) (Fig. 5E). Sulfinyl sulfone and thiosulfinate are capable for disproportionation [44], [45]. Presumably, the oxidation of DSF to its thiosulfinate derivative and further, at a later stage, to sulfinyl sulfone proceeds by a similar mechanism.

#### 3.3.4 Сytotoxicity of the products of the reaction between DDC and HOCbl

**Fig. 6:**
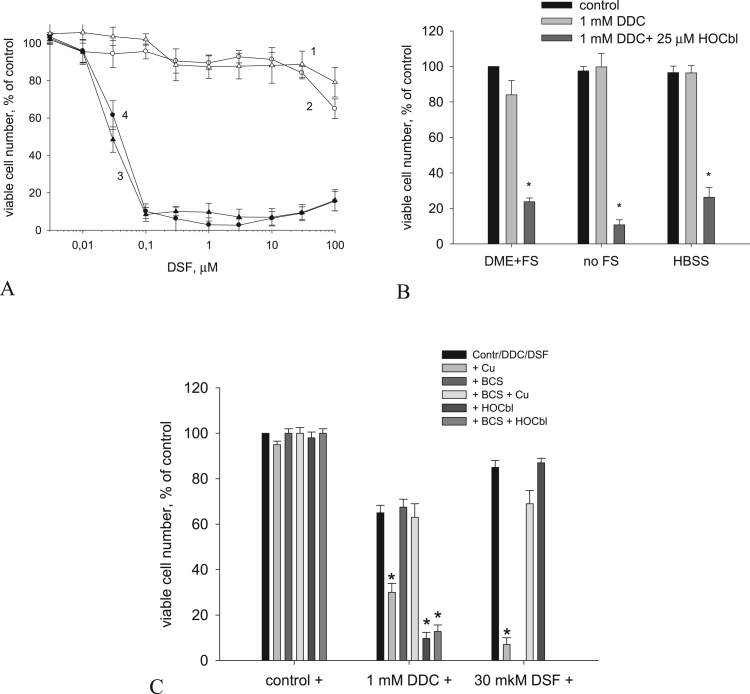
Cytotoxic effect of the products of the reaction of DDC + HOCbl in А549 cells. (А) Viability of А549 cells in the presence of the precipitate obtained in an aqueous solution of 10 mM DDC and 250 μM HOCbl (*1*) and of commercial DSF (*2*) in А549 cells without the addition of copper ions and after the addition of 4 μM CuSO_4_ (*3*, *4*, respectively). (В) Cytotoxicity of DDC + HOCbl in Cu-free and Cu-containing media. Incubation time: 3 h with serum-free DMEM and 6–7 h with DMEM + FS and HBSS. Then, incubation media were replaced by a fresh growth medium DMEM supplemented with 10% FS. The cytotoxic effect was estimated 48 h after the addition of DDC + HOCbl. (С) Comparison of the effect of the chelator of extracellular copper BCS on the cytotoxic effect of 1 mM DDC + 25 μM HOCbl and on the toxicity of 1 mM DDC/30 μM DSF + Cu^2+^ (4 μМ) in DMEM + FS. BCS (50 μM) was added 1 h prior to the addition of DDC + HOCbl and DDC/DSF + Cu^2+^. The cytotoxicity was estimated at 48 h after the addition of the components (see Materials and methods). The data are the means ± SEM of five separate experiments. *P < 0.01 compared to cells treated with 1 mM DDC or 30 mkM DSF.

We analyzed the cytotoxic effect of the products of the reaction of DDC with HOCbl: the precipitate obtained in the reaction of 10 mM DDC with 250 μM HOCbl and the supernatant obtained in the reaction of 1 mM DDC + 25 μM HOCbl. We also compared the cytotoxic effect of the precipitate with that of commercial DSF. It was found that both the precipitate and DSF were not toxic at concentrations approaching the limit of their solubility, which in DMEM medium supplemented with 10% FBS is 114.2 ± 26.4 μM. As known, copper ions significantly increase DSF toxicity. The addition of copper ions (2–4 μM CuSO_4_ which was nontoxic by itself) enhanced the cytotoxic effect of both the sediment and DSF in the growth medium with serum (Fig. 6A): the IC_50_ value for both DSF and the sediment decreased more than 1000 times, to 0.03–0.04 μM. HOCbl did not affect the toxicity of DSF. Thus, DSF, which is one of the products of the reaction of DDC with HOCbl, produced a cytotoxic effect in DMEM supplemented with serum only at concentrations above 100 μM, and for enhancing its cytotoxicity at concentrations less than 100 μM, the addition of 2–4 μM copper ions was needed.

It is known that, in the growth medium containing serum, copper ions can be present; they are capable of producing the cytotoxic effect due to their reaction with DDC or with the resulting DSF. Therefore, we determined whether the cytotoxicity of DDC + HOCbl takes place in solutions containing no copper ions, including media without serum and HBSS. For this purpose, 24 h after cell seeding, the growth medium in the wells was replaced either by serum-free DMEM or HBSS, after which DDC + HOCbl was added. After 3–6 h, the incubation solution was replaced with a fresh growth medium, and cell viability was estimated after 48 h. It was found that HOCbl enhanced cell death in the presence of 1 mM DDC in both DMEM without serum and in HBSS (Fig. 6B). The incubation of cells in these solutions with 1 mM DDC had no cytotoxic effect (Fig. 6B). In addition, the role of copper ions in the enhancement of the cytotoxicity of DDC + HOCbl was estimated using the chelator BCS, which binds extracellular copper ions. In the concentration range 50–500 μM, the chelator did not increase cell viability during the incubation with DDC + HOCbl (Fig. 6C) or with 1–3 mM DDC alone (Fig. 6C). At the same time, at relatively low concentrations of 30–50 μM, BCS successfully counteracted the cytotoxic effect induced by the addition of copper (2–10 μM) to cells in the presence of DDC or DSF (Fig. 6C). Thus, the cytotoxic effect of DDC + HOCbl did not depend on the presence of extracellular copper ions.

## 4 Discussion

**Fig. 7:**
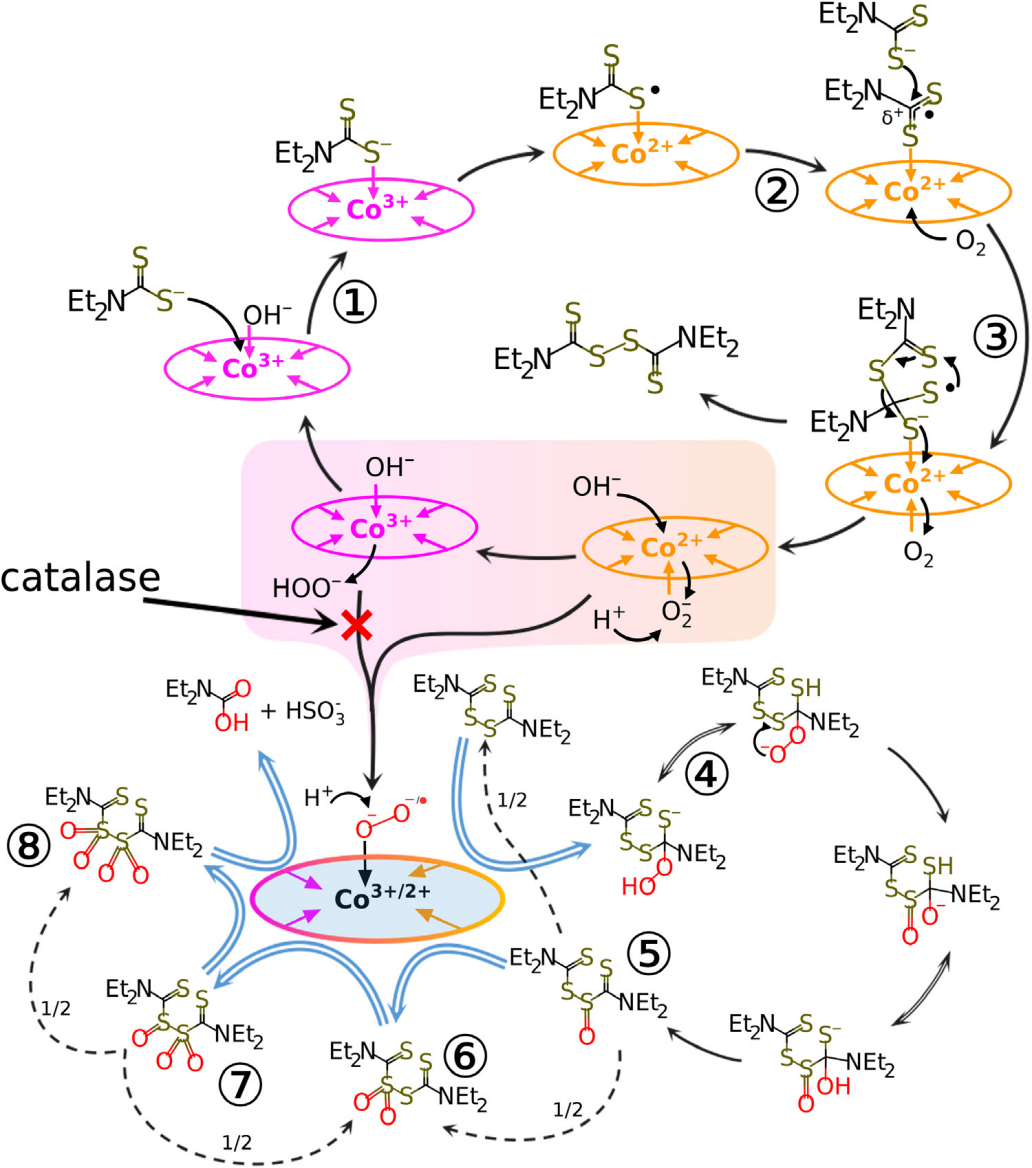
A scheme of the oxidation of DDC in the presence of HOCbl. The major transformations are denoted by a black solid line arrows; the disproportionation of unstable oxidized derivatives of DSF is shown by a black dashed line arrows; oxidation of DSF and its oxidized products, catalyzed by a complex Cbl–(HO_2_^-^/O_2_^2-^), is shown by a blue line double arrows; ①, ②, ③ – the stage of DDC oxidation; ② - the stage that is sensitive to thiols present in the system. ④ – A hypothetical mechanism of the oxidation DSF derivatives by the action of a cobalamin–hydroperoxide/superoxide anion complex; Left Right Double Arrows denote the reversible stage of the reaction, and blue double arrows denote the irreversible stage of the reaction; ⑤, ⑥, ⑦, ⑧ – the structures of oxidized DSF derivatives.

It is known that cobalamins are potent catalyzers of the oxidation of thiols by oxygen [6], [46], [47], which can lead to the accumulation of H_2_O_2_ in physiological solutions in toxic concentrations [7], [8]. As distinct from other thiols, DDC contains a thiocarbothionyl fragment capable of delocalizing the electron density [9], [10], which affects the properties of reaction products resulting from oxidation. In particular, despite the decrease in the oxygen level, we were unable to detect any traces of hydrogen peroxide in medium after the addition of DDC and HOCbl. It can be assumed that the oxidation of DDC in the presence of HOCbl occurs by the following scheme (Fig. 7). At the first stage, DDC interacts with HOCbl (Co(III)) to form a DDC–Cbl complex (Fig. 7, ①). As a result of the intramolecular redox reaction, the electron is transported from the sulfur atom to cobalt, with the unpaired electron being delocalized toward the thiocarbothionyl fragment. At the second stage, a second DDC molecule attacks the DDC–Cbl complex (Fig. 7, ②). The subsequent rearrangement and transport of the second electron to cobalt result in the formation of DSF. At this stage, the oxidation of DDC to DSF is inhibited by the administration of simple thiols. We believe that this effect is due to the competitive interaction of thiols with DDC. This interaction occurs at the stage of the electron transport from the sulfur atom of DDC to cobalt, which results in the formation of the intermediate Cbl (II)–DDC^•^ (Fig. 7, ②). Further interaction can proceed by the free-radical mechanism; namely, the DDC radical reacts with thiol to form a mixed disulfide of DDC and thiol, which is further oxidized at the sulfur atom of simple thiol (GSH or NAC). We have shown earlier that the thiols GSH and NAC in the reaction with HOCbl can enter into the redox reaction to form Н_2_О_2_, which leads to cell death. However, in the reaction system involving DDC + HOCbl, these thiols produce a protective effect, which also points to the formation of a mixed disulfide whose both components are inactive toward HOCbl. The formation of a mixed disulfide causes a significant decrease in the amount of both DSF (as demonstrated by experiments with the inhibition of precipitation of DSF crystals in the presence of GSH) and its oxidized forms, which leads to a partial inhibition of the cytotoxic effect.

The final stage of the formation of DSF is the transfer of the second electron from the sulfur atom to cobalt (Fig. 7, ③) and then to oxygen, which forms a coordinated bond on the other side of the HOCbl molecule. In these conditions, one could expect the production of hydrogen peroxide in solution. Nevertheless, this does not occur. The addition of catalase to the mixture leads only to a twofold decrease in the rate of uptake of dissolved oxygen. A similar effect was described in the literature [43]. The authors related the decrease in the reaction rate to the competitive reaction of oxidation of DSF to a disulfone derivative. The production and accumulation of hydrogen peroxide in solution can be hindered by the competitive reaction of oxidation of DSF to a disulfone derivative. That the accumulation of hydrogen peroxide does not occur in this reaction is also evidenced by the fact that the addition of catalase did not completely abolish the cytotoxic effect of the DDC + HOCbl mixture.

Presumably, further oxidation of DSF proceeds with the immediate involvement of the unstable HOCbl–superoxide anion complex whose presence was confirmed earlier [48].

Probably, the first stage of DSF oxidation is the binding of superoxide anion (or peroxide anion) to the carbon atom of the thiocarbothionyl fragment of DSF, which results in the formation of DSF peroxide (Fig. 7, ④). The peroxide can undergo an intramolecular rearrangement with the formation of a thiosulfinate derivative of DSF (Fig. 7, ⑤). This asymmetric compound can be oxidized further in a similar way. An alternative mechanism is also possible by which this sulfoxide enters the disproportionation reaction with the result that asymmetric thiosulfonate (Fig. 7, ⑥) and DSF form (denoted by dashed arrows). Presumably, the thiosulfonate and disulfone derivatives (Fig. 7, ⑥ and ⑧) of DSF can be oxidized only by the action of superoxide anion or hydrogen peroxide in the presence of HOCbl, whereas thiosulfinate and sulfinyl-sulfone (Fig. 7, ⑤ and ⑦) are capable of disproportionating to their more oxidized and reduced derivatives (indicated by dashed arrows). The final products of oxidation may be diethylcarbamic acid and sulfite anion. The formation of DSF oxidation products was recorded by us by mass spectrometry and was indirectly confirmed by an increase in light absorption at 210 nm, which is characteristic of sulfoxides.

Thus, our data indicate that the HOCbl-catalyzed oxidation of DDC by oxygen can result in the formation of DSF and its sulfone and sulfoxide derivatives. DSF is slightly soluble in aqueous solutions; according to the literature data, its solubility at 25 °С is 13 μM [49], and at this concentration it produced no cytotoxic effect in our experiments. We found that the thermodynamic solubility of DSF at 37 °С is 69–75 μM in PBS and HBSS and 114 μM in DMEM supplemented with serum. In a medium containing 1 mM DDC + 25 μM HOCbl, single DSF crystals were detected, indicating that the DSF solubility threshold is attained. During the 48-h incubation in culture medium with serum, DSF at these concentrations showed a weak cytotoxic effect (Fig. 6C). The cytotoxic effect of DSF was enhanced many times in the presence of copper ions [13], [14], [17], [18], [25], [26], [28], [30], which just was shown in our experiments with the addition of DSF in the presence of extracellular Cu^2+^ (2–4 μM). However, the enhancement of the cytotoxicity of DDC by HOCbl does not depend on the presence of copper ions in medium. Therefore, we assumed that the main role in the cytotoxic effect is played not by DSF itself, but by other reaction intermediates, soluble oxidized derivatives of DSF.

The cytotoxic effects of oxidized DSF derivatives are poorly understood, primarily due to their strong instability [50]. As known, sulfoxides and sulfones are amphiphilic and more polar compounds compared with sulfides. This is confirmed by the calculations of the coefficients of distribution (СlogP, ChemAxon) according to which the lipophilicity of DSF and its oxidized derivatives varies in the following order: DSF (4.16) > sulfoxide of DSF (3.50) > thiosulfonate of DSF (3.28) > sulfinyl sulfone of DSF (2.67) > disulfone of DSF (2.40). This suggests that active products are more effectively transported to targets inside the cell; in this case, sulfoxide derivatives do not react with GSH and hence are more stable than sulfones [51]. It has been shown earlier that the S-methyl sulfoxide derivative DETC-MeSO, a product of DSF bioactivation, is a selective and potent inhibitor that carbamoylates SH groups of enzymes, e.g., aldehyde dehydrogenase, with the effective concentration of the derivative being by one order of magnitude lower than that of DSF [13], [52], [53], [54], [55], [56], [57], [58]. It was shown that the sulfoxide derivative of the carbamate insecticide aldicarb is more toxic toward some mammalian and invertebrate species than the initial substance and the sulfonic derivative [59], [60]. These data suggest that the enhancement of the cytotoxicity of DDC caused by the addition of HOCbl could be due to the formation of the oxidized forms of DSF, sulfones and sulfoxides. A direct confirmation of this suggestion is problematic due high instability [50] of these compounds.

In summary, a 6 h exposure to DDC + HOCbl produces the cytotoxic action independently of extracellular Cu ions probably due to the accumulation of oxidized DSF derivatives. Thus, under in vitro conditions, DDC + HOCbl is a binary catalytic system with a prolonged toxic effect.
